# Knowledge of Shaken Baby Syndrome Among Polish Nurses and Midwives: A Cross-Sectional National Survey

**DOI:** 10.3390/children12091160

**Published:** 2025-08-31

**Authors:** Alina Głowińska, Sebastian Glowinski

**Affiliations:** 1 Independent Researcher, 74-024 Koszalin, Poland; alina.glowinska@o2.pl; 2 Institute of Health Sciences, Pomeranian University in Slupsk, Westerplatte 64, 76-200 Slupsk, Poland; 3 Institute of Physical Culture, The State Academy of Applied Sciences in Koszalin, Lesna 1, 75-582 Koszalin, Poland

**Keywords:** shaken baby syndrome, nurses’ knowledge, infant care, Poland, abuse prevention

## Abstract

**Highlights:**

- 94.5% of nurses had heard of Shaken Baby Syndrome (SBS), but only 5.5% received formal training;
- 27.3% experienced emotional overload while caring for a crying infant;
- Older nurses showed better knowledge of colic and safer coping strategies;
- 92.7% expressed interest in SBS and infant crying management training;
- Only 29.1% believed mothers are informed about SBS in maternity wards.

**Abstract:**

Background: Shaken Baby Syndrome (SBS) is a severe form of abusive head trauma with potentially fatal consequences. Nurses and midwives play a crucial role in its prevention through early recognition and caregiver education; however, little is known about their knowledge and preparedness in the Polish context. Objectives: To assess the knowledge, perceptions, and educational experiences related to SBS among Polish nurses and midwives working with infants. Participants and Setting: A nationwide cross-sectional survey was conducted among 110 nurses and midwives employed in neonatal and pediatric care settings across Poland. Methods: An anonymous questionnaire collected demographic data and evaluated knowledge of SBS, infant crying, coping strategies, and prior training. Associations between knowledge levels and participant characteristics were analyzed using the Mann-Whitney U and Kruskal-Wallis tests. Results: Most participants (94.5%) had heard of SBS, and 78.2% correctly recognized shaking as more dangerous than a fall from a changing table. However, only 5.5% reported receiving formal training on SBS. Recognition of SBS symptoms was generally high (e.g., vomiting 100%, seizures 90.9%), but misconceptions persisted regarding coping with infant crying. More than one-quarter (27.3%) admitted experiencing a “breaking point,” and this group was more likely to acknowledge the risk of losing emotional control. Older nurses demonstrated significantly better recognition of crying patterns and colic (*p* = 0.0415), while SBS knowledge was positively associated with years of professional experience (*p* = 0.0484). Conclusions: Although general awareness of SBS is widespread, practical knowledge and training remain insufficient. Structured educational programs on SBS and infant crying management are urgently needed to better prepare healthcare professionals and reduce the risk of caregiver-related harm to infants.

## 1. Introduction

Abusive Head Trauma (AHT), commonly referred to as Shaken Baby Syndrome (SBS), is a serious brain injury that results from violently shaking an infant or young child [1,2]. SBS is characterized by a set of neurological symptoms caused by such trauma, including intracranial hemorrhages, brain damage, and retinal injuries [3,4]. These injuries can lead to permanent and irreversible consequences—ranging from intellectual disabilities and motor impairments to vision problems and even death [5]. In addition to these neurological manifestations, SBS may also present with non-neurological clinical signs, including skin bruising—most often on the upper limbs—and, more rarely, fractures of the upper extremities [6,7]. For this reason, SBS is considered one of the most severe forms of infant abuse and remains a critical issue in both health prevention and pediatric care [8,9].

Understanding the causes, symptoms, mechanisms, and long-term consequences of SBS is essential for medical professionals—especially nurses, who work closely with infants and their families on a daily basis [10,11]. Due to their role in monitoring the health and development of young children, nurses are often in a unique position to recognize early signs of SBS. Moreover, they play a key role in educating parents and caregivers about safe ways to respond to infant crying and how to prevent violent behavior [12,13,14].

In Poland, as in many other countries, the issue of SBS remains insufficiently understood and inadequately addressed [15,16]. Despite numerous studies on child abuse and head trauma, there is a lack of specific data regarding Polish nurses’ knowledge of shaken baby syndrome. This knowledge is critically important, as failure to recognize symptoms promptly can delay appropriate diagnostic and therapeutic interventions, ultimately worsening a child’s health outcomes [17]. However, official data on the incidence or prevalence of SBS in Poland are not collected, which makes it difficult to determine the actual scale of the problem in the national context.

Moreover, the role of nurses extends beyond merely recognizing symptoms—they also play an important part in educating families [18,19]. In cases where parents or caregivers are unaware of the dangers associated with shaking a baby, proper education can serve as a key factor in preventing SBS [20]. In this context, it is essential that nurses possess not only medical knowledge, but also strong communication and teaching skills, enabling them to effectively convey information and offer support to parents [21].

In recent years, numerous initiatives have been launched in Poland to raise public awareness about child abuse and to promote safe caregiving practices for infants [22]. However, for these efforts to be truly effective, a thorough understanding of the current knowledge level among healthcare providers is also necessary. A lack of adequate education and awareness among nurses can lead to underestimating the risks or delaying both medical and social interventions.

Research on SBS has been ongoing for many years, examining the impact of shaking on a child’s health [5,23]. This includes the development of biomechanical models and experiments involving animals or dummies [15,24,25], which help estimate the forces and accelerations infants may be subjected to during such traumatic events [26,27,28].

This study presents a nationwide analysis of the level of knowledge about shaken baby syndrome among Polish nurses and midwives who work with infants under the age of one. The study has a cross-sectional design and aims not only to assess current knowledge but also to identify educational gaps and factors influencing familiarity with SBS. The findings may serve as a foundation for creating educational recommendations and training programs that will enhance the quality of infant care and contribute to more effective SBS prevention in Poland.

## 2. Materials and Methods

A prospective, cross-sectional, multicenter, anonymous, and confidential survey was conducted among nurses and midwives working in hospitals across Poland between 20 April and 5 July 2025. The target group comprised healthcare professionals who regularly care for infants up to one year of age. Participation was voluntary, and individuals who did not consent were excluded.

The survey invitation was first sent via email to hospital wards, and only after obtaining institutional approval was the survey link distributed to nursing staff. The questionnaire was developed based on existing literature and previously published surveys on SBS, infant crying, and caregiver coping strategies. Content validity was established through literature review and expert consultation, while face validity and clarity were assessed in a pilot group of nurses (n = 10). Items on SBS symptoms were derived from standard clinical descriptions, and questions on infant crying reflected the well-documented peak in crying frequency at around 6 weeks of age. Feedback from the pilot phase was incorporated into the final instrument. Two partially completed questionnaires were excluded, resulting in a final dataset of 110 female participants.

The study was conducted in accordance with the principles of the Declaration of Helsinki. Prior to data collection, approval to conduct the survey was obtained from the management of each participating hospital. Participation was entirely voluntary and anonymous. Respondents were informed about the aims of the study, assured that no personal identifiers would be collected, and reminded that they could withdraw at any stage without providing a reason. Completion and submission of the questionnaire were considered to imply informed consent. These procedures ensured that participants’ autonomy, confidentiality, and wellbeing were fully respected.

The survey tool consisted of three sections:

I. Section 1 covered general questions such as year of birth, work experience, average monthly working hours, education, specialization, workplace, and number of children.
II. Section 2 included 14 questions assessing knowledge about Shaken Baby Syndrome (SBS).
III. Section 3 focused on strategies for coping with a crying baby.

All analyses were performed using Statistica 13.3 software (TIBCO Software Inc., Palo Alto, CA, USA) [29]. The normality of quantitative data was assessed with the Shapiro–Wilk test, which is considered appropriate for modest sample sizes. Non-parametric tests (Mann-Whitney U, Kruskal-Wallis) were applied where assumptions of normality were not met, and parametric approaches (t-test) were used only when distributional assumptions were satisfied. Quantitative variables were described using the mean ($\bar{x}$), standard deviation (SD), median (Me), minimum (Min), maximum (Max), and coefficient of variation (V). Qualitative variables were presented in tables as absolute numbers and percentages. To evaluate relationships between quantitative variables, Pearson’s correlation coefficient (r) was calculated, and statistical significance was determined using the *p*-value.

## 3. Results

### 3.1. Respondent Demographics

A total of 110 nurses and midwives took part in the study. Table 1 provides descriptive statistics for the entire group, including the mean, standard deviation (SD), 95% confidence intervals (CI), median values, and ranges. The average age of participants was 46.25 years (SD = 12.44), with a median of 49 and an age span from 23 to 64 years. On average, participants had 22.17 years of professional experience (SD = 13.34), with a median of 24 and a range from 1 to 43 years. Their average monthly working time amounted to 158.83 h (SD = 28.45), with reported working hours ranging from 35 to 280 per month.

**Figure 1:**
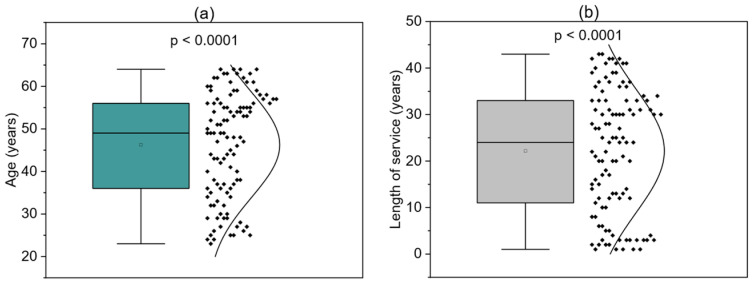
Box-plot of participants’ age (**a**) Box-plot of work experience (**b**).

In terms of family status, 41.82% of respondents had two children, while 25.45% reported having none. Figure 1a illustrates the age distribution of participants. The boxplot shows a median age close to 50, with the majority of values concentrated between approximately 35 and 60 years. The dot plot and the density curve further confirm this distribution, suggesting it approximates a normal distribution. The *p*-value (*p* < 0.0001) indicates a statistically significant result in relation to the tested hypothesis.

Figure 1b shows the distribution of years of professional experience. Again, the data are presented using a boxplot, dot plot, and density curve. The median length of service is around 24 years, with values extending up to 43 years. The *p*-value from the Shapiro–Wilk test (*p* < 0.0001) also points to statistical significance.

**Figure 2:**
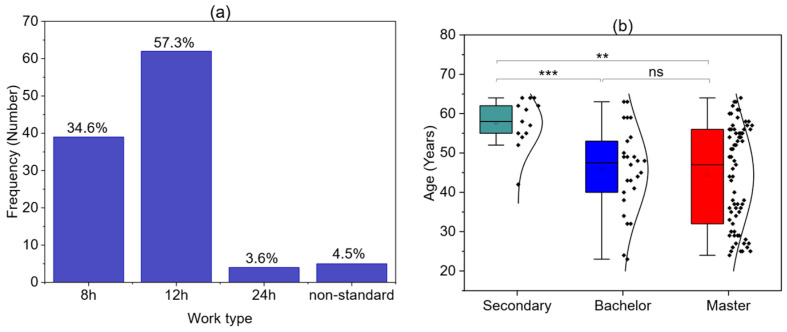
Type of work performed (**a**) Education (**b**): ** *p* < 0.01; *** *p* < 0.001; ns—non-significant.

The majority of respondents reported working 12-hour shifts (63 individuals—57.3%), while 38 participants (34.6%) worked fixed 8-hour shifts (Figure 2a). Only 4 participants (3.6%) reported working 24-hour shifts, and 5 nurses (4.5%) had irregular or non-standard working hours. In terms of education, 71 participants (64.5%) held a master’s degree, 26 (23.6%) had a bachelor’s degree, and 13 (11.8%) had completed secondary-level nursing education. As shown in Figure 2b, there is a statistically significant difference in age between nurses with secondary education—who were the oldest on average—and those holding bachelor’s or master’s degrees (*p* = 0.0009 Kruskal–Walli’s test). The analysis did not reveal a strong correlation between age, length of service, monthly working hours, and the number of children.

### 3.2. Respondents’ Knowledge of SBS

To better understand how much people know about Shaken Baby Syndrome (SBS), the survey included a set of questions aimed at exploring both general awareness and more specific beliefs about the condition. Respondents were asked whether they had ever heard of SBS, how they perceive its severity compared to other risks (such as a fall from a changing table), and when they first encountered the term.

The questionnaire also addressed perceptions about the frequency of SBS cases in Poland, possible symptoms, and long-term consequences. In addition, it explored opinions on who is most likely to shake a baby, what situations may lead to such behavior, and whether individuals—including the respondents themselves—might lose control in moments of extreme stress, such as dealing with persistent infant crying. This section presents the results of those responses, shedding light on the current state of knowledge and attitudes toward SBS among the participants.

Table 2 presents the responses to a set of questions assessing participants’ knowledge and awareness of Shaken Baby Syndrome (SBS), based on a sample of 110 individuals. The vast majority of respondents (94.54%) reported having heard of SBS. When asked about which scenario poses a greater danger to a child, 78.18% identified shaking as more hazardous compared to a fall from a changing table (21.82%). Regarding the point at which respondents first encountered the term “shaken baby,” answers varied: 35.45% learned about it during university studies, 30.91% during training sessions, and 29.09% in their professional practice. Only a small proportion (4.55%) stated they had never heard the term before. Most participants (84.55%) believed that cases of shaking are more prevalent in Poland than incidents involving falls from changing tables (15.45%). Estimates of the number of SBS cases occurring annually in Poland ranged widely, with the most common responses being 101–200 cases (23.64%) and more than 300 cases per year (26.36%).

When asked about the estimated percentage of children hospitalized due to shaking, 62.73% of respondents believed the figure to be between 0–9%, while 31.82% estimated it to fall within the 10–18% range. Finally, in response to whether they had encountered a case of shaking during their professional career, 17.27% had witnessed such a case personally, 10.91% knew of one through colleagues, and 37.27% had come across cases through media sources. Just over a third (34.55%) reported having no such encounters.

Table 3 summarizes participants’ responses regarding the clinical signs, consequences, and underlying causes associated with Shaken Baby Syndrome (SBS), based on the same group of 110 respondents. When asked about symptoms that may indicate a child has been shaken, the most frequently identified signs included emesis (100%), sleep disorders (96.36%), feeding difficulties (85.45%), irritability (88.18%), and loss of consciousness (89.09%). Other commonly recognized symptoms were pallor and cyanosis (64.55%), epileptic seizures (90.91%), and retinal abnormalities (81.82%). Notably, a high proportion also recognized motor impairment (94.55%) and somnolence (67.27%) as possible indicators. Regarding long-term outcomes, 35.45% of respondents estimated that between 21–30% of infants with SBS suffer from significant neurological and developmental disorders, while 30% believed the rate was up to 20%. Fewer respondents selected higher estimates: 22.73% for 31–40% and 11.82% for 41–50%. In terms of perpetrator perception, half of the respondents (50%) indicated that the main perpetrator of shaking was a cohabiting partner or concubine. Mothers were indicated by 32.73%, and fathers by 14.54%. Grandparents and caregivers were rarely selected. As for the most common triggers for shaking, increased crying was almost unanimously recognized (96.36%), followed by alcohol or drug use (95.45%), domestic violence (98.18%), stress at work or in marriage (79.09%), and financial pressures (43.64%). Mother’s depression was also acknowledged by 84.55% of respondents, and child illness by 40%. When asked whether every caregiver could be capable of shaking a baby due to prolonged crying, half of the participants (50%) believed there is such a possibility, while 26.36% responded that they didn’t think so. Only 6.36% were certain it could happen to anyone, and 12.73% rejected the idea entirely. A similar distribution was seen in responses to the question of whether anyone could lose control while caring for an inconsolably crying child. Almost half (47.27%) said it was possible, while 30% did not believe so. Just 11.82% were convinced it could happen, and 10.91% rejected the idea.

Finally, when asked about their own emotional responses, 72.73% of participants stated they had always kept calm when dealing with a crying baby, whereas 27.27% admitted to experiencing a situation they described as a “breaking point.”

### 3.3. Ways of Coping with a Crying Baby

Caring for a crying baby can be one of the most challenging experiences for parents and caregivers. It often brings feelings of stress, helplessness, and frustration, especially when the crying is prolonged or intense. Understanding how to effectively cope with this situation is crucial—not only to support the child’s needs but also to help prevent harmful reactions that may arise from overwhelming emotions. This section explores various aspects related to managing infant crying, based on the experiences and opinions of nurses, who play a key role in educating and supporting parents. The questions cover knowledge about infant crying, practical advice for parents, training related to coping strategies, and the communication of risks associated with shaking a baby. Through this, we aim to gain insight into how healthcare professionals perceive and contribute to this important aspect of infant care.

Table 4 outlines respondents’ knowledge and attitudes regarding infant crying, as well as their views on how parents should respond in challenging situations involving prolonged crying. The data were collected from 110 participants.

In the knowledge assessment section, 66.36% correctly recognized that babies tend to cry more often in the late afternoon or evening, and 58.18% were aware that crying typically intensifies in the first few weeks of life, peaking around two to three months before gradually subsiding. A large majority (80.91%) understood that healthy infants may cry without a clear reason, and an equal proportion acknowledged that crying does not always indicate pain. However, only 40% of respondents believed it was normal for infants to cry for more than two hours a day. Additionally, just 43.64% agreed that healthy babies can cry for several hours at a time, while the majority (56.36%) disagreed.

Notably, 68.18% did not agree that a parent can step away from their baby when the crying becomes frustrating, and 27.27% felt that a good parent should always be able to soothe a crying infant—suggesting persistent social pressure or guilt surrounding parenting roles.

When asked what a parent should do if their baby cried for a prolonged period while they were home alone, nearly all participants recommended soothing the baby (100%) and asking others for help (97.27%). Only a small minority considered calling an ambulance (24.55%) or placing the baby in another room (6.36%) to be appropriate. Just 14.55% supported staying in the same room while tuning out the crying with headphones.

Regarding how long a parent can remain in the same room with a crying child, responses varied: 27.27% selected up to 30 min, 23.64% up to an hour, and 4.55% up to two hours. Nearly half (44.55%) believed a parent might stay with the crying child continuously.

In the final section, addressing questions about infant colic, 90.91% of respondents agreed that colic can be a cause of prolonged crying lasting for several months. Similarly, 81.82% reported being able to recognize colic-related crying. Most respondents (95.45%) also acknowledged that colic can lead to parental stress, including feelings of irritability, exhaustion, or being overwhelmed.

Table 5 presents responses related to experiences with and attitudes toward receiving support and education about coping with crying infants and shaken baby syndrome (SBS), based on a sample of 110 participants. Only a small minority of respondents reported having previously participated in preventive training on coping with crying babies (6.36%) or on the topic of SBS (5.45%). However, there was a strong interest in such education: 92.73% expressed willingness to participate in training on SBS and on managing crying. Regarding current practices in maternity wards, fewer than half (47.27%) believed that mothers receive information about the difficulties of newborn care (such as fatigue, colic, crying, and sleep issues). Another 41.82% responded negatively, and 10.91% noted that there is simply no time for this kind of information to be conveyed.

The awareness of SBS in maternity settings appeared even lower—only 29.09% thought that mothers are informed about its dangers, while 65.45% said they are not, and 5.45% cited time constraints. When asked who should be responsible for providing parents with information on infant care challenges and associated risks, respondents widely endorsed multiple sources. The most frequently identified were: prenatal classes (97.27%), general practitioners (93.64%), maternity wards (90.91%) and midwives/private services (92.73%). Additionally, 77.67% pointed to family as an important source, and 69.09% emphasized support from the close social circle.

### 3.4. Factors Associated with SBS Knowledge and Perceptions

**Figure 3:**
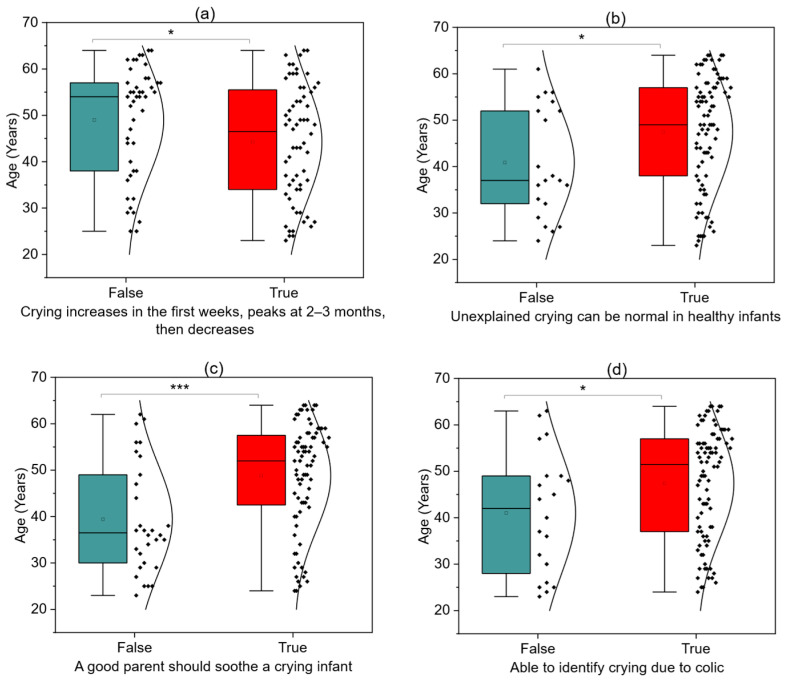
Crying typically increases during the first few weeks of life, peaks around 2–3 months, and then gradually subsides (**a**); A healthy infant may sometimes cry unexpectedly or without an apparent reason (**b**); A good parent should be able to soothe their crying baby (**c**); Ability to recognize crying caused by colic (**d**) *** *p* < 0.001; * *p* < 0.05.

Figure 3a illustrates the distribution of knowledge regarding infant crying across age groups. Younger respondents more often recognized that infant crying typically intensifies during the first few weeks of life, peaks around 2–3 months, and then gradually subsides (*p* = 0.411, UMW test). In contrast, older nurses were significantly more likely to agree that a healthy baby may sometimes cry unexpectedly without any obvious reason (Figure 3b, *p* = 0.0288, UMW). Moreover, they more frequently endorsed the belief that a good parent should be able to soothe their crying infant (Figure 3c, *p* = 0.0008, UMW). They were also more inclined to believe that if a baby cries for an extended period and the caregiver is alone at home, calling emergency services would be an appropriate course of action (Figure 3d, *p* = 0.0270, UMW). Finally, older respondents demonstrated a better ability to identify colic as a cause of persistent crying (Figure 3d, *p* = 0.0415, UMW test).

**Figure 4:**
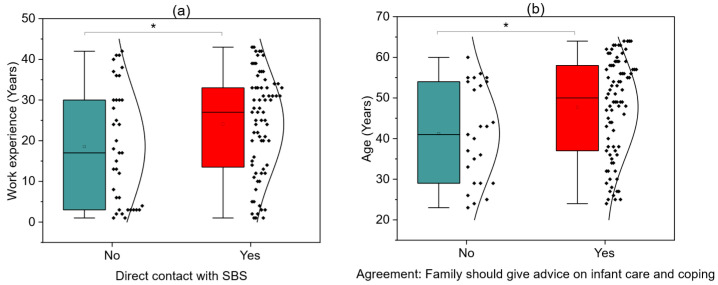
Have you encountered Shaken Baby Syndrome (SBS) during your professional career? (**a**); Should families receive information on how to cope with an infant in difficult situations? (**b**); * *p* < 0.05.

Figure 4a shows that respondents who reported direct contact with SBS (had significantly more years of work experience compared to those without such contact (*p* = 0.0484 UMW test). Figure 4b demonstrates that those who agreed that families should provide advice on infant care and coping in difficult situations were significantly older than those who disagreed (*p* = 0.0169 UMW test). These findings suggest that both greater professional experience and age may be associated with stronger recognition of the importance of family involvement in infant care education and coping strategies.

### 3.5. Breaking Point Situations and Factors Associated with Loss of Emotional Control

**Figure 5:**
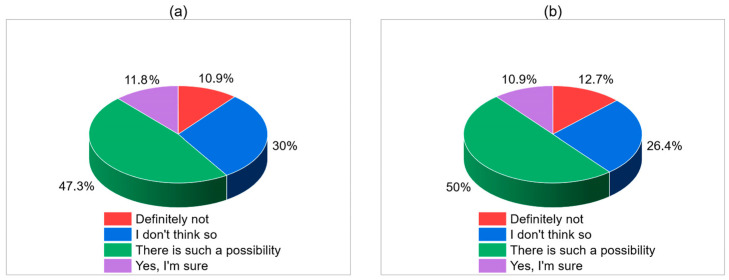
Can anyone lose control while caring for a crying baby (unsoothable, prolonged crying) (**a**); Is every caregiver capable of shaking a baby due to prolonged crying? (**b**).

Among the 110 surveyed nurses, 27.27% (n = 30) admitted experiencing a so-called “breaking point”—an extreme emotional situation during which they felt an urge to shake a crying infant to stop the crying. The remaining 72.73% (n = 80) declared that they had always remained calm in such situations. Initial comparison suggests that nurses who reported a breaking point were slightly younger on average than those who had never experienced such feelings. Moreover, they had fewer children, indicating that less parenting experience might be a risk factor for lower emotional resilience. Unfortunately, the observed differences were not statistically significant (*p* = 0.7145 UMW). A higher proportion of those who had a breaking point agreed with the statement that “anyone can lose control when exposed to prolonged infant crying,” suggesting greater awareness of the universality of emotional limits (Figure 5a,b). Younger respondents were more likely to strongly agree that it is possible to lose control when caring for a crying baby (prolonged inconsolable crying), compared to older respondents, who were more likely to strongly disagree. Interestingly, fewer of those who reported a breaking point had previously participated in training on Shaken Baby Syndrome (SBS). At the same time, they were more likely to express willingness to attend such training in the future, possibly reflecting increased awareness of their own vulnerability and need for support strategies.

## 4. Discussion

The findings of this study indicate that greater awareness of Shaken Baby Syndrome (SBS) and prior participation in SBS-related training are associated with safer and more constructive coping strategies when managing prolonged infant crying. Respondents with higher knowledge levels or training were less likely to endorse harmful practices, such as leaving the infant unattended or ignoring the crying, and more likely to adopt active, empathetic approaches, including seeking help, taking a safe break, or contacting medical services. Notably, participants with greater SBS awareness were significantly less likely to report experiencing a “breaking point”—a moment of emotional overload accompanied by the urge to shake a baby. This suggests that knowledge may serve as a protective factor against stress-induced impulsive behavior.

Almost all respondents (95%) recognized alcohol or drug use as a trigger for shaking, yet only half acknowledged that anyone might “break” under pressure. This discrepancy highlights a gap between theoretical knowledge and self-perceived emotional resilience. Moreover, although 93% of participants had never received formal SBS training, 92% expressed a strong interest in such education, emphasizing the urgent need for systematic training programs.

Another important finding concerns the relatively high average age of the surveyed nurses, reflecting the demographic profile of the pediatric and neonatal nursing workforce in Poland. With their greater professional experience and awareness of SBS risks, older nurses may play a pivotal role in transferring knowledge to younger colleagues. Beyond recognizing clinical symptoms, they can contribute to parental education on safe coping strategies for infant crying and help prevent abusive behaviors. Strengthening peer-to-peer knowledge exchange within nursing teams may represent a valuable, low-cost strategy to improve SBS prevention efforts at both clinical and community levels. Our analysis also distinguished between age and professional experience: age was mainly associated with recognition of infant crying patterns and colic, while years of practice correlated with SBS knowledge. This suggests that experiential learning acquired in clinical settings may be particularly important for SBS awareness, whereas broader life experience may support understanding of infant crying and coping strategies.

This study has several limitations. The sample was relatively small, self-selected, and non-random, which may reduce generalizability and introduce selection bias. The questionnaire, although based on existing literature and pilot-tested for clarity, was not subjected to full psychometric validation. These constraints are common in SBS-related research, where the phenomenon is both sensitive and relatively rare, and the present findings should therefore be interpreted as exploratory. Nevertheless, the sample size (n = 110) is comparable to other cross-sectional studies of healthcare professionals’ knowledge of SBS [30,31,32], and the nationwide scope—including nurses from different regions and care settings—provides contextual value. Still, representativeness remains uncertain, as participation was voluntary and may not reflect the actual distribution of healthcare professionals working with infants in Poland.

Finally, the absence of official epidemiological data on SBS in Poland makes it difficult to estimate the true scale of the problem. From a forensic perspective, one of the authors encountered two SBS-related cases over the past six years while serving as a court expert in human biomechanics, both of which were fatal. These experiences underscore the rarity but also the severity of SBS in the Polish context.

Despite these limitations, this study provides important preliminary evidence of knowledge gaps regarding SBS among Polish nurses and midwives. Larger, randomized studies with validated instruments are warranted to confirm these findings and build a more comprehensive understanding of SBS awareness among healthcare professionals in Poland.

## 5. Conclusions

This study highlights notable gaps in Polish nurses’ and midwives’ knowledge of Shaken Baby Syndrome and emphasizes the protective role of education in preventing harmful responses to infant crying. To address these gaps, several action points should be considered:

- Integrating mandatory SBS-related modules into undergraduate nursing and midwifery curricula.
- Offering regular continuing professional development sessions on SBS recognition, prevention, and parental education.
- Encouraging peer-to-peer mentoring within hospital units to facilitate knowledge transfer from more experienced to younger staff.
- Developing structured parental education programs that promote safe coping strategies for infant crying and raise awareness of the dangers of shaking.

By combining professional training with family education, healthcare systems can contribute to reducing the risk of SBS and improving the safety and wellbeing of infants.

## Figures and Tables

**Table 1:** Description statistics of the group (N = 110): Mean (SD), [95% CI], Median, Range.

Variable		Variable	
Age [years]	46.25 (12.44) [43.90; 48.61] 49 23–64	Working time per month [hours]	158.83 (28.45) [153.45; 164.20] 160 35–280
Length of service [years]	22.17 (13.34) [19.65; 24.69] 24 1–43	Number of children	0 children—28 (25.45%) 1 child—16 (14.55%) 2 children—46 (41.82%) 3 children—15 (13.64%) 4 and more—5 (4.55%)

**Table 2:** Answers to questions related to knowledge about SBS (N = 110) Part 1.

Questions	Answers	
Have you heard of the phenomenon of SBS?	Yes	104 (94.54%)
	No	6 (5.46%)
2. What is more dangerous for a child?	Shake	86 (78.18%)
	Falling off table	24 (21.82%)
3. When did you first encounter the term “shaken baby”?	At university	39 (35.45%)
	In training	34 (30.91%)
	At work	32 (29.09%)
	Not heard	5 (4.55%)
4. In your opinion, are there more cases in Poland:	Shake	93 (84.55%)
	Falling off table	17 (15.45%)
5. How many cases of shaken baby syndrome occur annually in Poland?	up to 30	7 (6.36%)
	from 31 to 60	18 (16.36%)
	from 61 to 100	20 (18.18%)
	from101 to200	26 (23.64%)
	from 201 to300	10 (9.09%)
	>300	29 (26.36%)
6. What percentage of children are hospitalized as a result of shaking?	up to 9%	69 (62.73%)
	from 10 to 18%	35 (31.82%)
	from 19 to 40%	5 (4.55%)
	from 41 to 50%	1 (0.91%)
7. Have you encountered a case of shaking a child during your professional career?	Yes, personally	19 (17.27%)
	From colleagues	12 (10.91%)
	From media (TV, Internet)	41 (37.27%)
	No	38 (34.55%)

**Table 3:** Answers to questions related to knowledge about SBS (N = 110) Part 2.

Questions	Answers	
8. Symptoms indicating that the child was shaken	Irritability	Yes 97 (88.18%), No 13 (11.82%)
	Somnolence	Yes 74 (67.27%), No 36 (32.73%)
	Feeding difficulties	Yes 94 (85.45%), No 16 (14.55%)
	Fretful and crying	Yes 92 (83.64%), No 18 (16.36%)
	Emesis	Yes 100 (100.00%), No 0 (0.00%)
	Pallor and cyanosis	Yes 71 (64.55%), No 39 (35.45%)
	Sleep disorders	Yes 106 (96.36%), No 4 (3.64%)
	Epileptic seizures	Yes 98 (89.09%), No 12 (10.91%)
	Loss of consciousness	Yes 98 (89.09%), No 12 (10.91%)
	Retinal abnormalities	Yes 90 (81.82%), No 20 (18.18%)
	Motor impairment	Yes 104 (94.55%), No 6 (5.45%)
9. What percentage of infants with shaking injury suffer from long-term and significant neurological and developmental disorders?	up to 20%	33 (30.00%)
	from 21 to 30%	39 (35.45%)
	from 31 to 40%	25 (22.73%)
	from 41 to 50%	13 (11.82%)
10. The main perpetrator of shaking a baby is:	Cohabitant/Concubine	55 (50.00%)
	Mother	36 (32.73%)
	Father	16 (14.54%)
	Grandmother/Grandfather	2 (1.81%)
	Caregiver	1 (0.91%)
11. The main trigger for shaking a baby is	Increased crying	Yes 106 (96.36%), No 4 (3.64%)
	Financial situation	Yes 48 (43.64%), No 62 (56.36%)
	Stress at work/marriage	Yes 87 (79.09%), No 23 (20.91%)
	Child’s illness	Yes 54 (49.09%), No 56 (50.91%)
	Alcohol/drugs/stimulants	Yes 105 (95.45%), No 5 (4.55%)
	Domestic violence	Yes 108 (98.18%), No 2 (1.82%)
	Mother depression	Yes 93 (84.55%), No 17 (15.45%)
12. Is every person caring for a child capable of shaking them due to prolonged crying?	Yes, I’m sure	7 (6.36%)
	There is such a possibility	55 (50.00%)
	I don’t think so	29 (26.36%)
	Definitely not	14 (12.73)
13. Do you think anyone can lose control when caring for a crying (unsoothed, prolonged) baby?	Yes, I’m sure	13 (11.82%)
	There is such a possibility	52 (47.27%)
	I don’t think so	33 (30.00%)
	Definitely not	12 (10.91%)
14. As a parent of a baby who cried for a long time, have you ever felt like shaking them to calm them down in an extreme situation?	I always kept calm	80 (72.73%)
	There was such a “breaking point” situation	30 (27.27%)

**Table 4:** Answers to questions related to Coping with a Crying Infant (N = 110).

Questions	Answers	
Assessment of knowledge about crying	Babies cry more often in the late afternoon or evening.	True 73 (66.36%); False 37 (33.64%)
	Infant crying intensifies in the first few weeks of life, peaks around 2–3 months, and then decreases.	True 64 (58.18%); False 46 (41.82%)
	A healthy baby sometimes cries unexpectedly or without an obvious reason.	True 89 (80.91%); False 21 (19.09%)
	Do you think it is normal for infants to cry more than 2 h a day?	True 44 (40.00%); False 66 (60.00%)
	Sometimes a crying baby may seem to be in pain, even though they are not.	True 89 (80.91%); False 21 (19.09%)
	Sometimes healthy infants can cry for several hours a day.	True 48 (43.64%); False 62 (56.36%)
	You can step away from a crying baby when their crying becomes frustrating.	True 35 (31.82%); False 75 (68.18%)
	A good parent should be able to soothe their crying baby.	True 80 (72.73%); False 30 (27.27%)
2. What do you think a parent should do if their baby cried for a long time and they were home alone?	Soothe the crying baby, hold them in your arms	True 110 (100.00%); False 0 (0.00%)
	Ask another person for help—relatives, parents, in-laws.	True 107 (97.27%); False 3 (2.73%)
	Call an ambulance.	True 27 (24.55%); False 83 (75.45%)
	Put the crying baby in the crib and go to another room.	True 7 (6.36%); False 104 (93.64%)
	Put the crying baby in the crib, stay in the same room (wear headphones and let the baby cry).	True 16 (14.55%); False 94 (85.45%)
3. How long do you think a parent can stay in the same room with a crying baby [h]?	up to 0.5 h	30 (27.27%)
	from 0.5 to 1 h	26 (23.64%)
	from 1 to 2 h	5 (4.55%)
	all the time	49 (44.55%)
4. Questions about infant colic	Can infant colic be the cause of prolonged crying lasting even several months?	Yes 100 (90.91%); No 10 (9.09%)
	Can you recognize crying caused by colic in a baby?	Yes 90 (81.82%); No 20 (18.18%)
	Can infant colic cause a parent to feel irritable, exhausted, or overwhelmed?	Yes 105 (95.45%); No 5 (4.55%)

**Table 5:** Answers to questions related to copying with a crying baby (N = 110).

Questions		
5. Have you participated in a preventive training on how to cope with crying babies?		Yes 7 (6.36%); No 103 (93.64%)
6. Have you participated in a preventive training on the topic of shaken baby syndrome?		Yes 6 (5.45%); No 104 (94.54%)
7. Would you like to participate in training on the topic of shaken baby syndrome and gain information on how to cope with a crying baby?		Yes 102 (92.73%); No 8 (7.27%)
8. Do you think that a mother in the maternity ward receives information about the difficulties that may arise from caring for a newborn (fatigue, colic, crying, the baby’s sleep, etc.)?		Yes 52 (47.27%); No 46 (41.82%) There is no time for that 12 (10.91%)
9. Do you think that mother in the maternity ward receives information about the dangers of SBS?		Yes 32 (29.09%); No 72 (65.45%) There is no time for that 6 (5.45%)
10. In your opinion, who should provide parents with information about the potential risks related to infant care and how to cope in difficult situations?	Maternity ward	Yes 100 (90.91%); No 10 (9.09%)
	Prenatal class	Yes 107 (97.27%); No 3 (2.73%)
	General practitioner (GP)	Yes 103 (93.64%); No 7 (6.36%)
	Close circle	Yes 76 (69.09%); No 34 (30.91%)
	Family	Yes 85 (77.67%); No 35 (22.73%)
	Midwife/private service	Yes 102 (92.73%); No 8 (7.27%)

## Data Availability

Article data are available upon request.

## References

[1] Thakir E.M., Jumaah L.F., Younis M.I. (2023). Evaluation of Mothers Knowledge about Shaken Baby Syndrome. J. Popul. Ther. Clin. Pharmacol..

[2] Vichon M. (2017). Shaken baby syndrome: What certainty do we have?. Child’s Nerv. Syst..

[3] Narang S.K., Estrada C., Greenberg S.D.L. (2016). Acceptance of Shaken Baby Syndrome and Abusive Head Trauma as Medical Diagnoses. J. Pediatr..

[4] Squier W. (2024). Retinodural haemorrhage of infancy, abusive head trauma, shaken baby syndrome: The continuing quest for evidence. Dev. Med. Child. Neurol..

[5] Glowinski S., Majdanik S., Glowinska A., Majdanik E. (2020). Trauma in a shaken infant? A case study. Aggress. Violent Behav..

[6] Feld K., Ricken T., Feld D., Helmus J., Hahnemann M., Schenkl S., Muggenthaler H., Pfeiffer H., Banaschak S., Karger B. (2022). Fractures and skin lesions in pediatric abusive head trauma: A forensic multi-center study. Int. J. Leg. Med..

[7] Pierce M.C., Kaczor K., Aldridge S., O’Flynn J., Lorenz D.J. (2010). Bruising characteristics discriminating physical child abuse from accidental trauma. Pediatrics.

[8] Irazuzta J.E., McJunkin J.E., Danadian K., Arnold F., Zhang J. (1997). Outcome and cost of child abuse. Child Abus. Negl..

[9] Ward M.G., Bennett S., King W.J. (2004). Prevention of shaken baby syndrome: Never shake a baby. Paediatr. Child Health.

[10] Coody D., Brown M., Montgomery D., Flynn A., Yetman R. (1994). Shaken baby syndrome: Identification and prevention for nurse practitioners. J. Pediatr. Health Care.

[11] Walls C. (2006). Shaken baby syndrome education: A role for nurse practitioners working with families of small children. J. Pediatr. Health Care.

[12] Barr R.G., Barr M., Fujiwara T., Conway J., Catherine N., Brant R. (2009). Do educational materials change knowledge and behaviour about crying and shaken baby syndrome? A randomized controlled trial. CMAJ.

[13] Fortin V., Ortiz A.R.A., Marq A.D., Mostermans E., Marichal M., Bailhache M. (2024). Childminder knowledge of shaken baby syndrome and the role played by childminders in prevention: An observational study in France. Arch. Pediatr..

[14] Zeifman D.M., St James-Roberts I. (2017). Parenting the Crying Infant. Curr. Opin. Psychol..

[15] Glowinski S., Glowinska A. (2025). Unveiling the Abusive Head Trauma and Shaken baby Syndrome: A Comprehensive Wavelet Analysis. Biomed. Signal Process. Control..

[16] Marcinkowska U., Paniczek M., Ledwoń M., Tyrała K., Skupnik R., Jośko-Ochojska J. (2015). Zespół dziecka potrząsanego w świadomości rodziców i personelu medycznego w Polsce. Pediatr. Pol..

[17] Kwiatkowski S., Franczak Young A., Kwiatkowska K. (2019). Neurological and psychological effects in Shaken Baby Syndrome. Szt. Leczenia.

[18] Altman R., Canter J., Patrick P.A., Daley N., Butt N.K., Brand D.A. (2011). Parent education by maternity nurses and prevention of abusive head trauma. Pediatrics.

[19] Smith K.M., DeGuehery K.A. (2008). Shaken baby syndrome education program: Nurses making a difference. MCN Am. J. Matern. Child. Nurs..

[20] Jakieła K., Curyl K. (2011). Udział pielęgniarki w rozpoznawaniu syndromu dziecka maltretowanego, przyjętego na oddział chirurgii dziecięcej. Pielęgniarstwo Chir. I Angiol.Surg. Vasc. Nurs..

[21] Bechtel K., Le K., Martin K.D., Shah N., Leventhal J.M., Colson E. (2011). Impact of an educational intervention on caregivers’ beliefs about infant crying and knowledge of shaken baby syndrome. Acad. Pediatr..

[22] National Campaign for a Violence-Free Childhood. https://www.polskieradio.pl/395/7789/artykul/3433258,polish-parliament-launches-campaign-to-combat-child-abuse-and-raise-awareness-against-violence.

[23] Mian M., Shah J., Dalpiaz A., Schwamb R., Miao Y., Warren K., Khan S. (2014). Shaken Baby Syndrome: A review. Fetal Pediatr. Pathol..

[24] Bonnier C., Mesples B., Gressens P. (2004). Animal models of shaken baby syndrome: Revisiting the pathophysiology of this devastating injury. Pediatr. Rehabil..

[25] Finnie J.W., Blumbergs P.C., Manavis J., Turner R.J., Helps S., Vink R., Byard R.W., Chidlow G., Sandoz B., Dutschke J. (2012). Neuropathological changes in a lamb model of non-accidental head injury (the shaken baby syndrome). J. Clin. Neurosci..

[26] Duhaime A.C., Gennarelli T.A., Thibault L.E., Bruce D.A., Margulies S.S., Wiser R. (1987). The shaken baby syndrome. A clinical, pathological, and biomechanical study. J. Neurosurg..

[27] Lloyd J., Willey E., Galaznik J., Lee I.W., Luttner S. (2011). Biomechanical evaluation of head kinematics during infant shaking versus pediatric activities of daily living. J. Forensic Biomech..

[28] Lee-Confer J.S., Wayman L.T., Havens K.L. (2025). High G-Forces in Unintentionally Improper Infant Handling: Implications for Shaken Baby Syndrome Diagnosis. Forensic Sci..

[29] www.statsoft.com.

[30] Alabdullah A.A.S., Ibrahim H.K., Aljabal R.N., Awaji A.M., Al-Otaibi B.A., Al-Enezi F.M., Al-Qahtani G.S., Al-Shahrani H.H., Al-Mutairi R.S. (2024). Awareness of Shaken Baby Syndrome among Saudi Nursing Students: A Cross-Sectional Study. Healthcare.

[31] Muhammad M.A., Shaker N.Z., Aziz G.K. (2021). Knowledge of Shaken Baby Syndrome among Hospital Nurses in Erbil City. Erbil J. Nurs. Midwifery.

[32] Allen K. (2014). The neonatal nurse’s role in preventing abusive head trauma. Adv. Neonatal Care.

